# Supplementary material for: Probiotic Properties and Proteomic Analysis of Pediococcus pentosaceus 1101
Source: Foods. 2022 Dec 22;12(1):46. doi: 10.3390/foods12010046 (PMC9818561; doi:10.3390/foods12010046)
Supplement: Supplementary file 1 [file foods-12-00046-s001.zip › foods-2083140-supplementary.pdf]

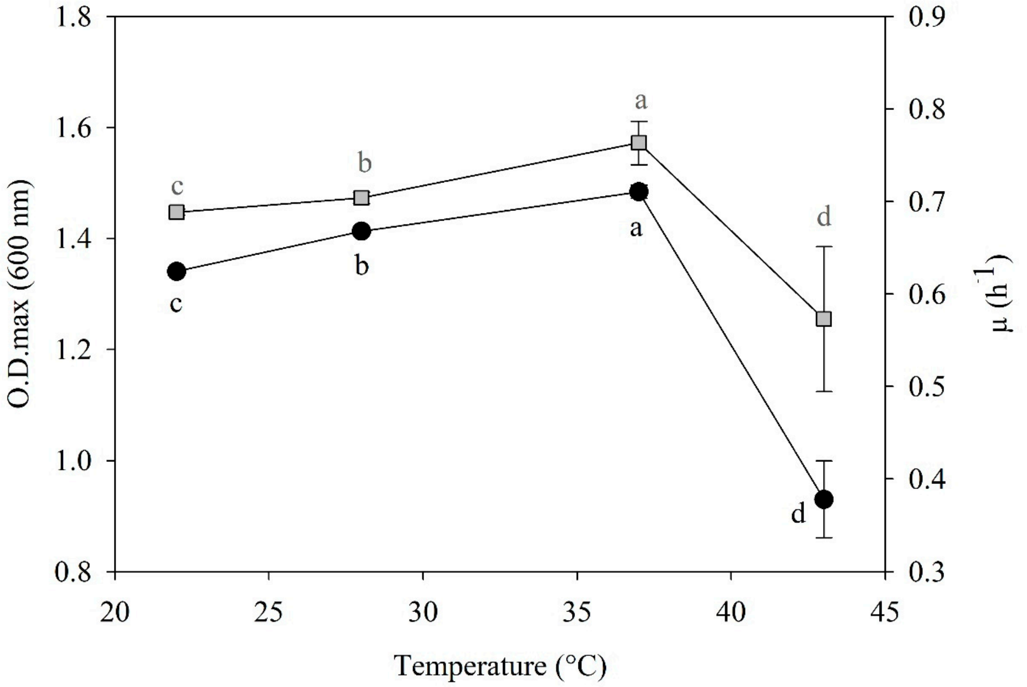

Figure S1. Influence of temperature (22, 28, 37 and 43 °C) on the growth of *P. pentosaceus* 1101: O.D. max (●), μ (■).The determinations were performed in triplicate, and the values represented are the mean with standard deviation of three independent experiments performed with three different cultures. Different letters indicate significant differences ($p < 0.05$) according to Duncan's multiple comparison of means.

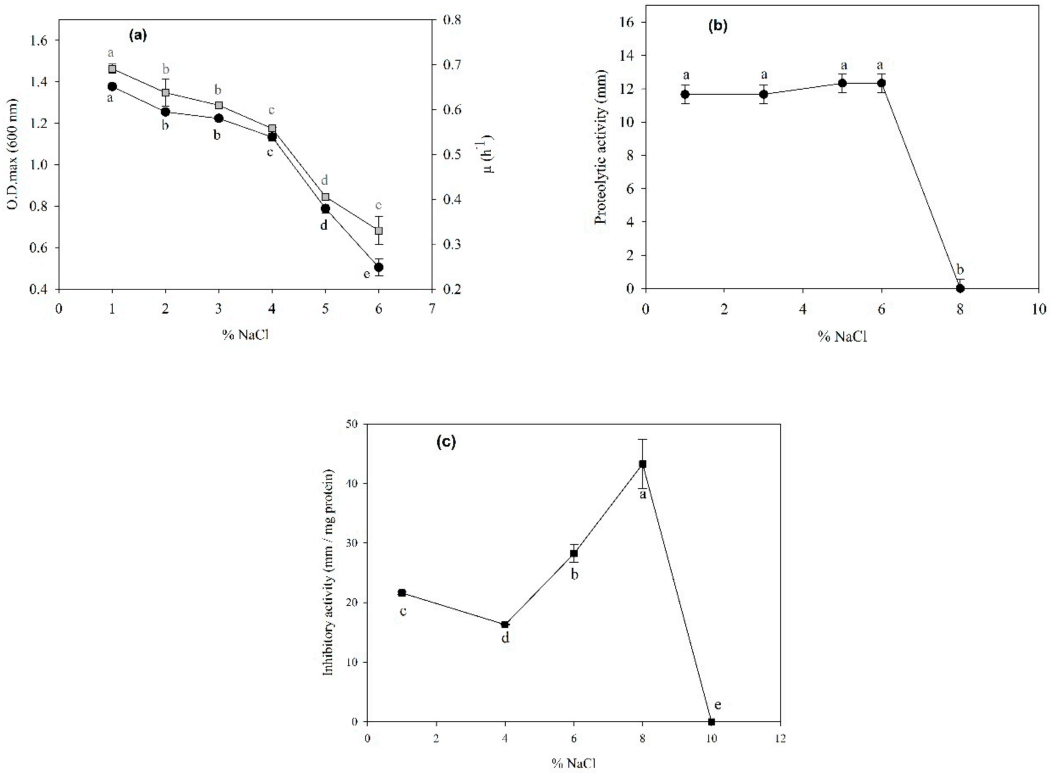

Figure S2. (**a**) Influence of NaCl (%) (1-6) on the growth of *P. pentosaceus* 1101: O.D. max (●), μ (■). (**b**) Proteolytic activity curve (% NaCl 1-8). (**c**) Inhibitory activity with *Listeria innocua* (% NaCl 1-10). The determinations were performed in triplicate, and the values represented are the mean with standard deviation of three independent experiments performed with three different cultures. Different letters indicate significant differences ($p < 0.05$) according to Duncan's multiple comparison of means.
